# Analysis of Ankle Muscle Dynamics during the STS Process Based on Wearable Sensors

**DOI:** 10.3390/s23146607

**Published:** 2023-07-22

**Authors:** Kun Liu, Shuo Ji, Yong Liu, Chi Gao, Shizhong Zhang, Jun Fu, Lei Dai

**Affiliations:** School of Mechanical and Aerospace Engineering, Jilin University, Changchun 130025, China; jishuo20@mails.jlu.edu.cn (S.J.);

**Keywords:** sEMG, STS, wearable sensor, muscle dynamics, inverse dynamics

## Abstract

Ankle joint moment is an important indicator for evaluating the stability of the human body during the sit-to-stand (STS) movement, so a method to analyze ankle joint moment is needed. In this study, a wearable sensor system that could derive surface-electromyography (sEMG) signals and kinematic signals on the lower limbs was developed for non-invasive estimation of ankle muscle dynamics during the STS movement. Based on the established ankle joint musculoskeletal information and sEMG signals, ankle joint moment during the STS movement was calculated. In addition, based on a four-segment STS dynamic model and kinematic signals, ankle joint moment during the STS movement was calculated using the inverse dynamics method. Ten healthy young people participated in the experiment, who wore a self-developed wearable sensor system and performed STS movements as an experimental task. The results showed that there was a high correlation (all R ≥ 0.88) between the results of the two methods for estimating ankle joint moment. The research in this paper can provide theoretical support for the development of an intelligent bionic joint actuator and clinical rehabilitation evaluation.

## 1. Introduction

The sit-to-stand (STS) movement is one of the common movements of human motion. Estimations of muscle and bone movements during the STS process can help doctors to better assess the health of patients and can also provide theoretical support for the development of an intelligent bionic joint assistor [1]. Muscle force and joint moment during the STS movement are an important basis for evaluating patients’ physical health. The Hill muscle model is the most common method to calculate muscle force; however, there are few studies on further calculation of joint moment during the STS movement after calculating muscle force [2,3]. Lower limb joint moment, especially ankle joint moment, is an important indicator for evaluating STS stability of human motion [4]. Ankle joint moment is the result of the action of muscle force and muscle moment arm, and to determine ankle joint moment it is necessary to calculate muscle force and muscle moment arm based on an appropriately established model. To calculate muscle force, there are many different muscle models for muscle force analysis, among which the Hill muscle model is the most ubiquitous muscle model [5,6]. Then, the muscle moment arm should also be calculated, which is the distance between a muscle path and the corresponding joint rotation center. To calculate the muscle moment arm, convenient musculoskeletal information of the ankle joint is necessary to show the relative positions of the ankle joint rotation center, muscle attachment point, and bone, by turning the ankle joint into a visual three-dimensional model. Numerous models for musculoskeletal information of the ankle joint have been developed at different fidelity levels to display the ankle joint [7]. However, there have also been some problems in these models, such as the active muscles responsible for ankle flexion only containing the tibialis anterior, neglecting the extensor digitorum longus, which leads to poor simulation modeling [8,9]. The method of calculating joint moment by calculating the muscle force and muscle moment arm is called forward dynamics.

In addition to the forward dynamics method, the inverse dynamics method is also commonly used for calculating joint moment during human movement. For example, based on human kinematics data and ground reaction, knee joint moments in the sagittal and frontal planes during human walking have been calculated using the inverse dynamics method [10]. Based on the data of human upper limbs motion, the joint moments of the elbow and shoulder joints have been calculated using the inverse dynamics method [11]. Based on a seven-limb model of the human body, hip, knee, and ankle joint moments during human walking have been calculated using the inverse dynamics method [12]. Based on data during the human jumping motion, the hip joint moment during human jumping has been calculated using the inverse dynamics method [13]. In this paper, the inverse dynamics method is used to calculate ankle joint moment during the STS movement. 

The forward and inverse dynamics methods for calculating ankle joint moment both require sensors to obtain human motion information, and it is common to use wearable sensors to obtain human motion information. In the past few years, wearable sensors have achieved significant advantages in obtaining human motion information. They can obtain various types of human motion information, such as inertial information and sEMG information, and wearable sensors are small in size, diverse in types, convenient, and comfortable to [14,15,16]. For example, human sweat contains many important biomarkers such as electrolytes, metabolites, and protein, which can be monitored by using wearable sensors in real time, and the changes in these parameters can be analyzed to provide an important basis for a clinician’s diagnosis [17,18]. A gait analysis based on a wearable sensor system also plays an important role in medical rehabilitation and clinical diagnosis [19]. In addition, an active pulse sensing system that could detect the weak vibration patterns of the human radial artery has been constructed with a sandwich-structure piezoelectret that had high equivalent piezoelectricity [20]. Based on motion data obtained using wearable sensors during human motion, the dynamic stability of the human body has been evaluated through a deep learning method [21]. Given that there are so many great examples and advantages of wearable sensors, a wearable sensor system should be self-developed for analysis of ankle muscle dynamics during the STS process using collected human motion information.

Moreover, there are studies in the literature that have demonstrated calculation of ankle joint moment through inverse dynamics or the study of ankle joint musculoskeletal models alone. Consequently, the main contribution of this study is to establish a musculoskeletal model of the ankle joint that includes more groups of muscles that contribute to the STS movement and to calculate ankle joint moment during the STS motion using the forward dynamics method and the inverse dynamics method. A self-developed wearable sensor system is used to obtain the sEMG and kinematic signals of the lower limbs during the STS movement. Using a Hill-based muscle model and based on the ankle musculoskeletal information, the muscle forces and muscle moment arms related to the ankle joint are calculated, and then the ankle joint moment is calculated. Finally, ankle joint moment is calculated using an inverse dynamics method to verify the accuracy of ankle joint moment calculated using the forward dynamics method. Through non-invasive means, the results provide a basis for doctors to evaluate patients’ physical conditions.

## 2. Method

**Figure 1:**
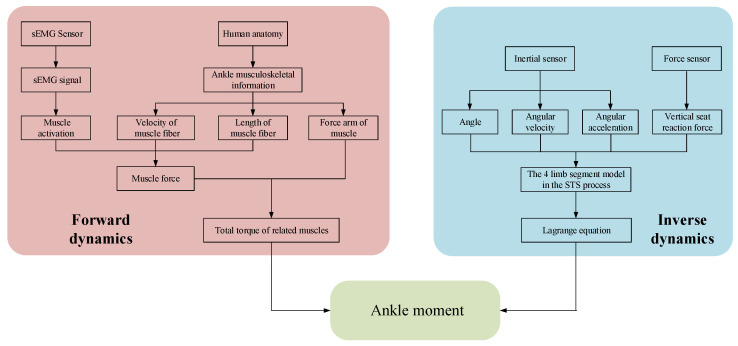
Logical flowchart for calculating joint moment using different methods.

A self-developed wearable sensor system made of sEMG sensors, inertial sensors, and force sensors was used to collect sEMG signals and kinematic signals on the lower limbs and vertical reaction force on the seat. Then, based on the established ankle joint musculoskeletal information and sEMG signals, the ankle joint moment during the STS process was estimated, as shown on the left side of Figure 1. Meanwhile, based on the established four-segment STS dynamic model and the kinematic signals and vertical seat reaction force, the ankle joint moment during STS movement was once more calculated using the inverse dynamics method, as shown on the right side of Figure 1. Eventually, the two different methods achieved the same ankle joint moment through different paths, and the accuracy of ankle joint moment was verified by comparing the two methods with each other.

### 2.1. Forward Dynamics Calculation of Joint Moment

#### 2.1.1. Activation Dynamics Models

In this paper, a linear activation dynamic model was adopted, where the root mean square (*RMS*) was used to predict muscle activation as follows:

(1) $$RMS=\sqrt{\frac{1}{N}(\sum_{i=1}^{N}v_{i}{}^{2})}$$

where *N* and *v_i_* are the number of sampling points and the voltage value of the *i*-th sampling point, respectively.

In this paper, the frequency of the sEMG signal sensor was 1000 Hz. The data during the whole STS movement were divided into several segments with 0.05 s as the time window based on the STS duration. The root mean square value of the data within each time window was calculated to provide a basis for calculating muscle activation. The length of the time window should be determined as needed, since short time windows can improve accuracy but also significantly increase computational complexity, while long time windows can effectively reduce computational complexity but also reduce accuracy. The sEMG signal derived from the sensor system were rectified, filtered, and divided into segments, on average, in the time domain. Then, the muscle activation of each segment was solved using Formula (2):

(2) $$a(i)=\frac{RMS(i)}{RMS_{\mathrm{MVC}}}$$

where *a(i)* is the muscle activation of segment *I*; *RMS(i)* is the *RMS* of the sEMG signal of segment *I*; and *RMS*_MVC_ is the *RMS* of the sEMG signal at the maximum voluntary contraction (MVC) of the muscle, which is normalized between 0 and 1.

#### 2.1.2. Muscle Tendon Model

**Figure 2:**
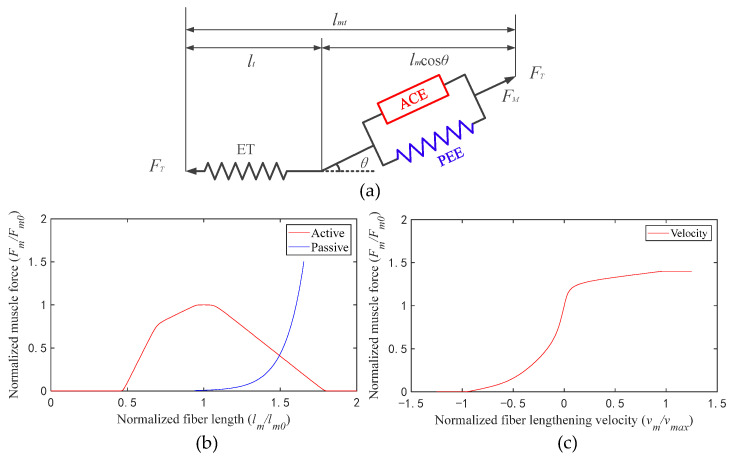
(**a**) Schematic of the muscle tendon model (*l_mt_* is the muscle length and *l_t_* is the tendon length); (**b**) active and passive force–length curves; (**c**) force–velocity curve.

A muscle tendon element was structured based on the Hill muscle model, which includes an active contractile element (ACE), a passive elastic element (PEE), and an elastic tendon (ET) (Figure 2a) [22]. The isometric properties of muscle are represented by an active contractile element (ACE) in parallel with a passive elastic element (PEE). The isometric muscle force is assumed to be the sum of muscle force when it is inactive (passive) and when it is excited (active). The muscle is in series with the tendon, which is represented by a nonlinear elastic element. The active tension develops when the nervous system excites muscle [23,24]. The active force that a muscle can generate varies nonlinearly with its length, represented by the active force–length curve (Figure 2b, active, red line). The passive force is generated when the muscle is stretched beyond a threshold length, whether the muscle is activated or not, which is represented by the passive force–length curve (Figure 2b, passive, blue line). During non-isometric contractions, the force generated by muscle varies nonlinearly with its rate of lengthening, which is represented by the force–velocity curve (Figure 2c, velocity, red line).

In order to calculate muscle fiber force conveniently, the curves were fitted into formulas. Formula (3) is the fitting formula of the active force–length curve (active, solid red line in Figure 2b); Formula (4) is the fitting formula of the passive force–length curve (passive, solid blue line in Figure 2b); Formula (5) is the fitting formula of the force–velocity curve (velocity, solid red line in Figure 2c):

(3) fACE(lm)=FmFm0=∑i=15bi·exp(-((lmlm0-ci)/di)^2)

where *f*_ACE_(*l_m_*) is the function of the fitted curve active, *F_m_* is the real-time active force of muscle fiber, *F*_*m*0_ is the peaking force of muscle fiber, *l_m_* is the real-time length of muscle fiber, *l*_*m*0_ is the optimum length of muscle fiber, where *b*_1_ = 10.39, *b*_2_ = −9.785, *b*_3_ = 0.8545, *b*_4_ = 0.3125, *b*_5_ = 0.3284, *c*_1_ = 0.8466, *c*_2_ = 0.8481, *c*_3_ = 1.117, *c*_4_ = 0.6376, *c*_5_ = 1.479, *d*_1_ = 0.1576, *d*_2_ = 0.1546, *d*_3_ = 0.2614, *d*_4_ = 0.1108, and *d*_5_ = 0.1976.

(4) $$f_{\mathrm{PEE}}(l_{m})=\frac{F_{P}}{F_{m0}}=2.342\cdot 10^{-6}\cdot \exp(8.085\cdot \frac{l_{m}}{l_{m0}})$$

where *f*_PEE_(*l_m_*) is the function of the fitted curve passive, *F*_P_ is the real-time passive force of muscle fiber, *F*_*m*0_ is the peaking force of muscle fiber, *l_m_* is the real-time length of muscle fiber, and *l*_*m*0_ is the optimum length of muscle fiber.

(5) f(vm)=FmFm0=∑j=17ej·exp(-((vmvmax-fj)/hj)^2)

where *f*(*v_m_*) is the function of the fitted curve velocity, *F_m_* is the real-time active force of muscle fiber, *F*_*m*0_ is the peaking force of muscle fiber, *v_m_* is the real-time velocity of muscle fiber, and *v_max_* is the max velocity of muscle fiber; its value is equal to 10 times the optimal fiber length, where *e*_1_ = 1.283, *e*_2_ = −21.66, *e*_3_ = −6.873, *e*_4_ = 0.8195, *e*_5_ = −0.6551, *e*_6_ = 0.08876, *e*_7_ = 26.84, *f*_1_ = 1.242, *f*_2_ = 0.2219, *f*_3_ = 0.3008, *f*_4_ = 0.3125, *f*_5_ = 0.1152, *f*_6_ = 1.36, *f*_7_ = 0.2371, *h*_1_ = 0.801, *h*_2_ = 0.1248, *h*_3_ = 0.1208, *h*_4_ = 0.6103, *h*_5_ = 0.08606, *h*_6_ = 1.36, and *h*_7_ = 0.1361.

When the muscle length changes, its pennation angle will also change. Real-time pennation angle values are as follows:

(6) $$\theta_{m}=\arcsin\left(\frac{l_{m0}\sin\theta_{0}}{l_{m}}\right)$$

where *θ_m_* is the real-time pennation angle, *θ*_0_ is the pennation angle at the optimum muscle fiber length, *l_m_* is the real-time length of muscle fiber, and *l*_*m*0_ is the optimum length of muscle fiber.

As shown in Figure 2, there is a pennation angle between tendon and muscle fiber, and the muscle output force is equal to the tendon force. The muscle output force is solved as follows:

(7) $$F_{T}=F_{\mathrm{ET}}=\left(F_{\mathrm{ACE}}+F_{\mathrm{PEE}}\right)\cdot \cos\theta_{m}=(a\cdot f_{\mathrm{ACE}}(l_{m})\cdot f(v_{m})+f_{\mathrm{PEE}}(l_{m}))\cdot F_{m0}\cdot \cos\theta_{m}$$

where *F*_T_ represents the output force of muscle, *F*_ET_ represents the force of tendon, *F*_ACE_ represents the active force of muscle fiber, *F*_PEE_ represents the passive force of muscle fiber, *a* is the muscle activation, and *θ_m_* represents real time pennation angle.

The muscle force in the STS sagittal plane is an important factor to solve the ankle joint moment, and the formula is as follows:

(8) $$F_{S}=F_{T}\cdot \cos\varphi_{m}$$

where *F*_S_ represents the muscle force in the STS sagittal plane, *F*_T_ represents the output force of muscles, and *φ_m_* represents the angle between the connecting line of the attachment points of muscles at both ends of ankle joint and the STS sagittal plane.

#### 2.1.3. Musculoskeletal Information

**Figure 3:**
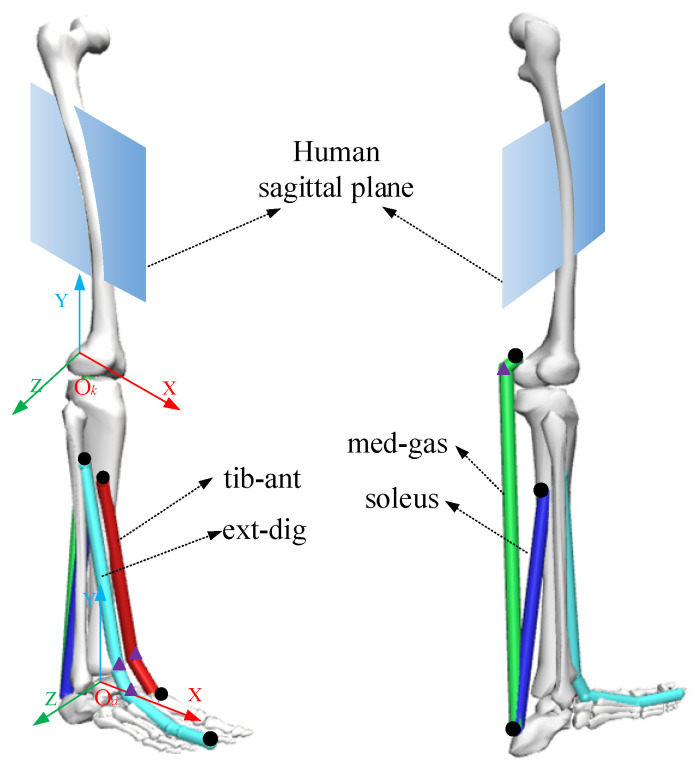
The musculoskeletal information of an ankle joint. The black dots represent the origin or insertion of muscles and the triangles represent intermediate “via” points.

According to human anatomy [25], the established ankle musculoskeletal information included the tibialis anterior (tib-ant), extensor digitorum longus (ext-dig), medial gastrocnemius (med-gas), soleus, and bones, as shown in Figure 3. Each muscle-tendon unit was represented by a line segment approximating the path of the anatomical muscle volume from the origin to the insertion, and when necessary, intermediate “via” points could be added for the calculated muscle moment arm and muscle length, as shown in Figure 3. At the same time, coordinate systems of the ankle joint (*O_k_-XYZ*) and knee joint (*O_a_-XYZ*) were established, with the rotation centers of the ankle and knee joints as origins. 

As shown in Figure 3, the moment arms of the muscle forces against the ankle joint in the STS sagittal plane (*XO_a_Y* plane) were determined using the Hill formula as follows:

(9) $$\begin{array}{l} a=\sqrt{{\left(x_{\mathrm{ap}1}-x_{\mathrm{ankle}}\right)}^{2}+{\left(y_{\mathrm{ap}1}-y_{\mathrm{ankle}}\right)}^{2}}\\ b=\sqrt{{\left(x_{\mathrm{ap}2}-x_{\mathrm{ankle}}\right)}^{2}+{\left(y_{\mathrm{ap}2}-y_{\mathrm{ankle}}\right)}^{2}}\\ c=\sqrt{{\left(x_{\mathrm{ap}2}-x_{\mathrm{ap}1}\right)}^{2}+{\left(y_{\mathrm{ap}2}-y_{\mathrm{ap}1}\right)}^{2}}\\ p=\left(a+b+c\right)/2\\ S=\sqrt{p\cdot \left(p-a\right)\cdot \left(p-b\right)\cdot \left(p-c\right)}\\ h=2\cdot S/p \end{array}$$

where *x*_ap1_, *y*_ap1_, *x*_ap2_, and *y*_ap2_ represent the coordinates of the projection points of muscle attachment points on both sides of the ankle joint in the *XO_a_Y* plane; *x*_ankle_ and *y*_ankle_ represent the projection point coordinates of the rotation center of the ankle joint in the *XO_a_Y* plane; *a, b,* and *c* represent the side lengths of the triangle formed by three projection points; *p* represents the variable; *S* represents the area of the triangle; and *h* represents the muscle moment arm in the sagittal plane of the human body.

#### 2.1.4. Joint Moment

In the *XO_a_Y* plane, the ankle joint moment acting by each muscle is calculated using Equation (10):

(10) $$M=F_{\mathrm{S-tib}}\cdot h_{\mathrm{tib}}+F_{\mathrm{S-ext}}\cdot h_{\mathrm{ext}}-F_{\mathrm{S-med}}\cdot h_{\mathrm{med}}-F_{\mathrm{S-soleus}}\cdot h_{\mathrm{soleus}}$$

where *M* represents the ankle joint moment; *F*_S-tib_, *F*_S-ext_, *F*_S-me_*,* and *F*_S-soleus_ represent the projection of muscle force of the tib-ant, ext-dig, med-gas, and soleus in the *XO_a_Y* plane; and *h*_tib_, *h*_ext_, *h*_med_, and *h*_soleus_ represent the moment arms of the tib-ant, ext-dig, med-gas, and soleus in the *XO_a_Y* plane.

### 2.2. Inverse Dynamics Calculation of Joint Moment

**Figure 4:**
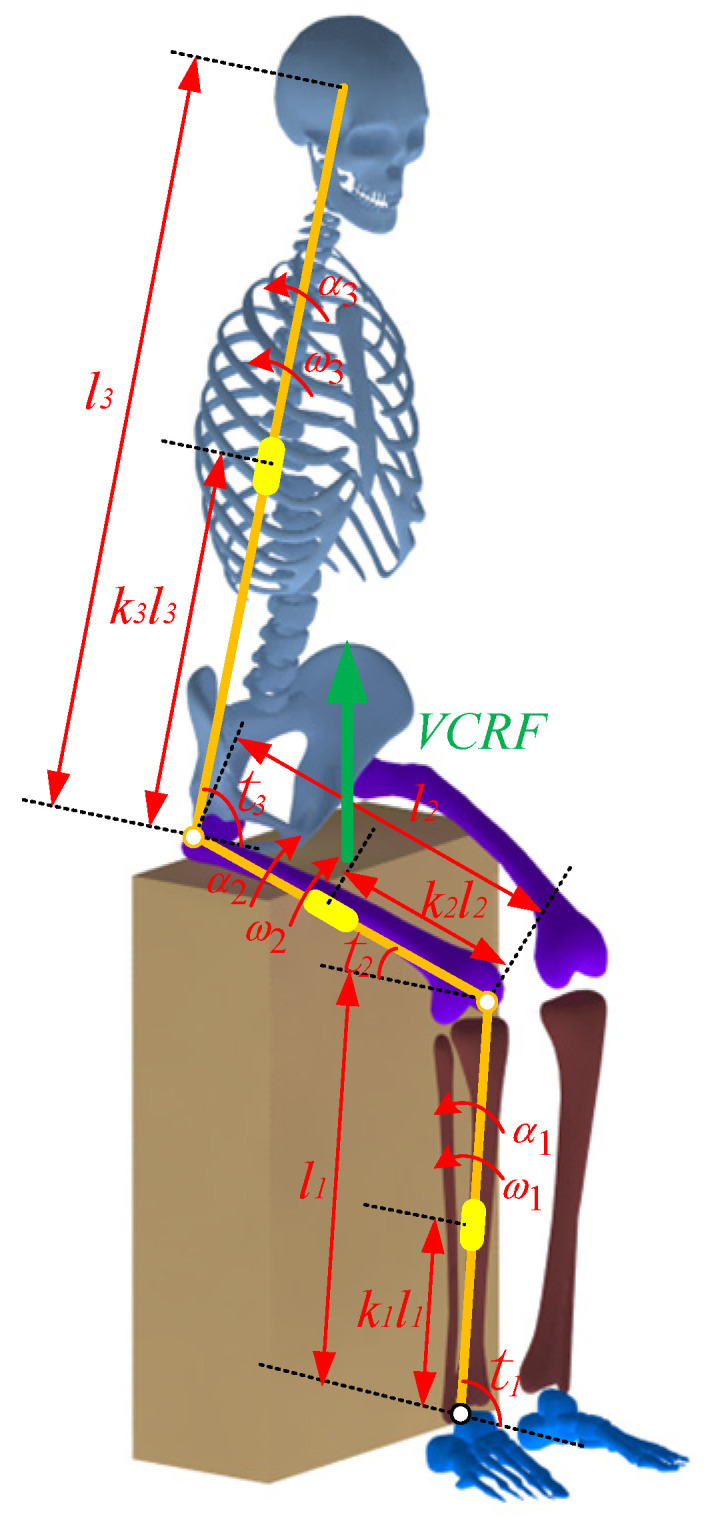
A four-segment STS dynamic model of the human body.

A four-segment STS dynamic model of the human body is established according to the movement of each body segment, as shown in Figure 4. Then, the ankle joint moment was calculated using the inverse dynamics method, as shown by Formulas (11) and (12):

(11) $$\begin{array}{l} n=\frac{F_{chair}}{G}\\ q=\frac{F_{chair}-n\cdot m_{2}g}{m_{3}g} \end{array}$$

(12) $$\begin{array}{cc} M= & (1-n)\cdot m_{2}g\left(l_{1}\cos t_{1}-k_{2}l_{2}\cos t_{2}\right)+(1-q)\cdot m_{3}g\left(l_{1}\cos t_{1}-l_{2}\cos t_{2}+k_{3}l_{3}\cos t_{3}\right)\\ & +\alpha_{1}\left[l_{1}{}^{2}\cdot \left(m_{1}k_{1}{}^{2}+m_{2}+m_{3}\right)+J_{1}\right]+\alpha_{2}l_{1}l_{2}\cdot \left(m_{2}k_{2}+m_{3}\right)\cdot \cos(t_{1}+t_{2})\\ & +\alpha_{3}m_{3}k_{3}l_{1}l_{3}\cdot \cos(t_{1}-t_{3})+k_{1}l_{1}m_{1}g\cos t_{1}\\ & -\omega_{2}{}^{2}\left(m_{2}k_{2}+m_{3}\right)l_{1}l_{2}\sin(t_{1}+t_{2})+\omega_{3}{}^{2}m_{3}k_{3}l_{1}l_{3}\cdot \sin(t_{1}-t_{3}) \end{array}$$

where *M* is the ankle joint moment; *J_i_*(*i* = 1, 2, 3) is the moment of inertia of the shank, thigh, and HAT (head, neck, arms, and torso) about the center of mass, respectively; *θ_i_*(*i* = 1, 2, 3) is the angle of the ankle, knee, and hip joint, respectively; *α_i_*(*i* = 1, 2, 3) is the angular acceleration of the ankle, knee and hip joint, respectively; *ω_i_*(*i* = 1, 2, 3) is the angular velocity of the ankle, knee, and hip joint, respectively; *k_i_*(*i* = 1, 2, 3) is the position factor of the center of mass of the shank, thigh, and HAT, respectively; *m_i_*(*i* = 1, 2, 3) is the mass of the shank, thigh, and HAT, respectively; *n* is the proportion of the thigh loading on the chair; *q* is the proportion of the HAT loading on the chair; *F_chair_* is the vertical-chair-reaction-force (VCRF); *G* is the gravity of the human body.

## 3. Experiment

In terms of sEMG signals, some studies have shown that there is no significant difference between men and women [26,27]. However, compared to women, men experience greater muscle strength and cross-sectional area during motions [28]. In experiments, larger muscle cross-sectional areas make it easier to find more accurate, expected active muscle positions, allowing for accurate attachment of sEMG sensors to collect corresponding sEMG signals. Therefore, in order to preliminarily verify the feasibility of the proposed method, only ten healthy young males (age = 25 ± 5 years, mass = 70 ± 10 kg, height = 170 ± 10 cm) were selected as the experimental subjects. The subjects had no lower limb musculoskeletal or neurological dysfunction, and could independently complete the STS movement. The experimental protocol was approved by the Human Ethical Review Committee of the First Hospital of Jilin University (No. 2023-221). Written and verbal instructions of testing procedures were provided, and written consent was obtained from the subjects prior to testing. 

**Figure 5:**
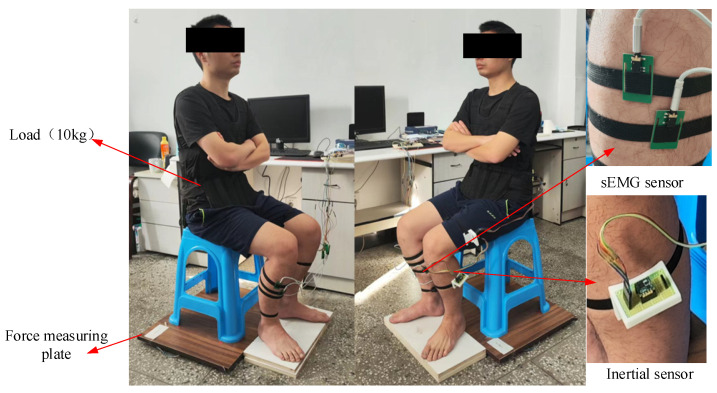
The experimental platform using the wearable multi-source sensing system.

An experimental prototype using inertial sensors (JY-901B, 100 Hz), sEMG sensors (Sizhirui, 1000 Hz), pressure sensors (YZC-1B, 100 Hz), and a STM32 microprocessor (Shenzhen Canyijia Electronic Technology Co., Ltd., Shenzhen, China) was self-developed. In order to measure the sEMG signals of the ankle muscles during the STS movement, 4 sEMG sensors (Sizhirui) were attached to the skin surfaces of the tib-ant, ext-dig, med-gas, and soleus muscle bellies using elastic straps, with 1000 Hz sampling frequency. In order to measure the angles, angular velocities, and angular accelerations of the human limbs during the STS movement, 3 inertial sensors (JY-901B, 100 Hz) were attached to the HAT, thigh, and shank using elastic straps at the position coinciding with the center of mass of each body segment in the sagittal plane, as shown in Figure 5. In order to measure the VCRF during the STS movement, a force measuring plate was developed using pressure sensors (YZC-1B, 100 Hz) located under the hip and feet. Finally, three different sensor-collected signals were simultaneously transmitted to the host STM32 microprocessor (Shenzhen Canyijia Electronic Technology Co., Ltd., Shenzhen, China) for offline processing and analyzing.

In order to increase the amplitude of the sEMG signal that reflects the muscle force related to the ankle joint, in the STS experiment, the subject had a waist load of 10 kg. In the initial state, the subject was seated with his upper body upright, arms folded in front of the chest, and feet freely placed; afterwards, the subject completed the STS movement.

## 4. Results

According to the peak force, the optimal fiber length, the tendon length, the pennation angle, and the attachment point position of muscle in the literature [29,30,31], the relevant parameters of the ankle joint muscles of the subjects were obtained by corresponding scaling. The experiment involved multiple subjects. First, Subject 1 (age = 26 years, mass = 73 kg, height = 170 cm) was used as an example to demonstrate the results analysis process. The ankle and knee joint coordinate systems of Subject 1 are shown in Figure 3. The muscle attachment point coordinates of the experimental subjects are determined based on their height and the length of their limb segments, so the attachment point coordinates of each experimental subject are different. To accurately describe the position of the muscle attachment point, the position of the muscle attachment point in the knee joint coordinate system is transformed into the ankle joint coordinate system through coordinate transformation. For Subject 1, the coordinates of the origin of his knee joint coordinate system in the ankle joint coordinate system during standing posture are (−11.2, 386, 0). The parameters related to the ankle muscles of Subject 1 are calculated and shown in Table 1. 

According to the human dimensions of Chinese adults and the inertial parameters of the adult human body, the mass, size, center of mass, and moment of inertia of each body segment of Subject 1 can be calculated, as shown in Table 2 [32,33].

**Figure 6:**
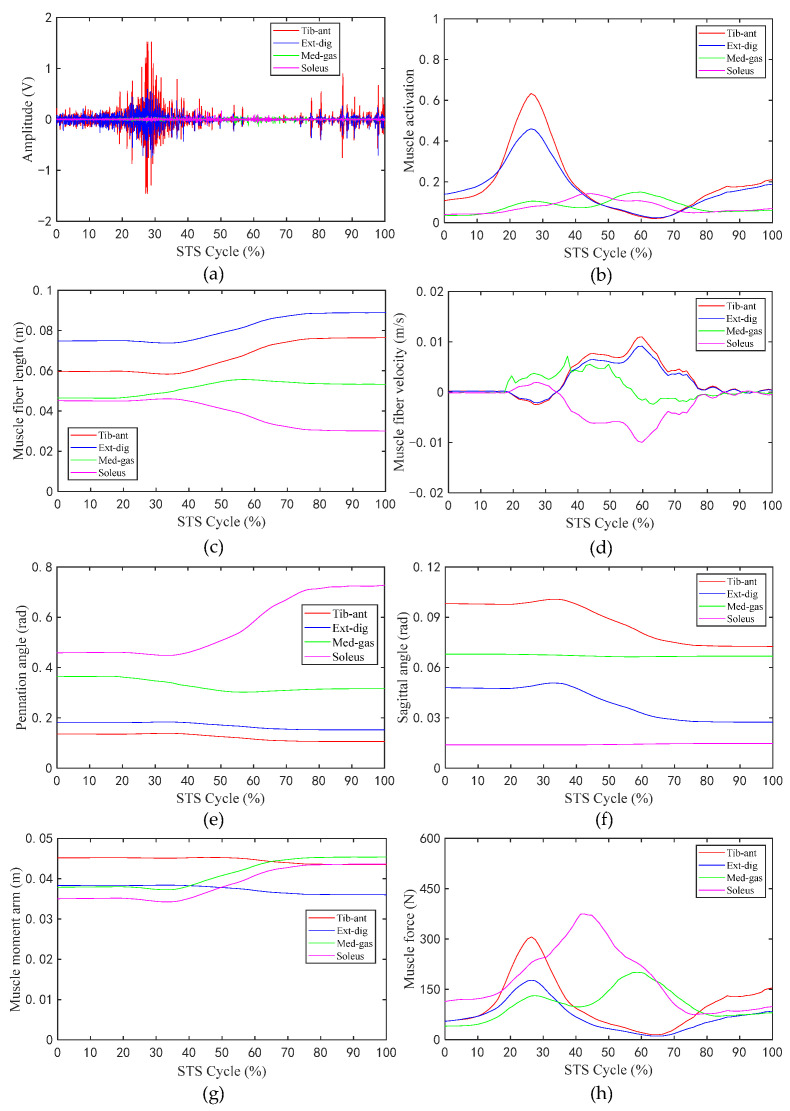
The values of related parameters of muscles during the STS process: (**a**) Amplitude of sEMG signal; (**b**) activation of muscle; (**c**) muscle fiber length; (**d**) muscle fiber velocity; (**e**) pennation angle of muscle; (**f**) sagittal angle of muscle; (**g**) muscle moment arm; (**h**) muscle force.

**Figure 7:**
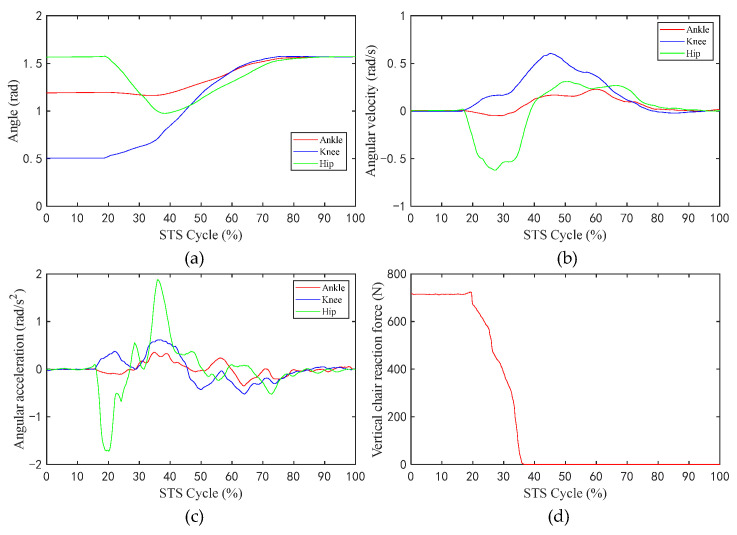
The average values of the angle, angular velocity, angular acceleration of the hip/knee/ankle, and VCRF, during the STS process: (**a**) Angle; (**b**) angular velocity; (**c**) angular acceleration; (**d**) vertical chair reaction force.

**Figure 8:**
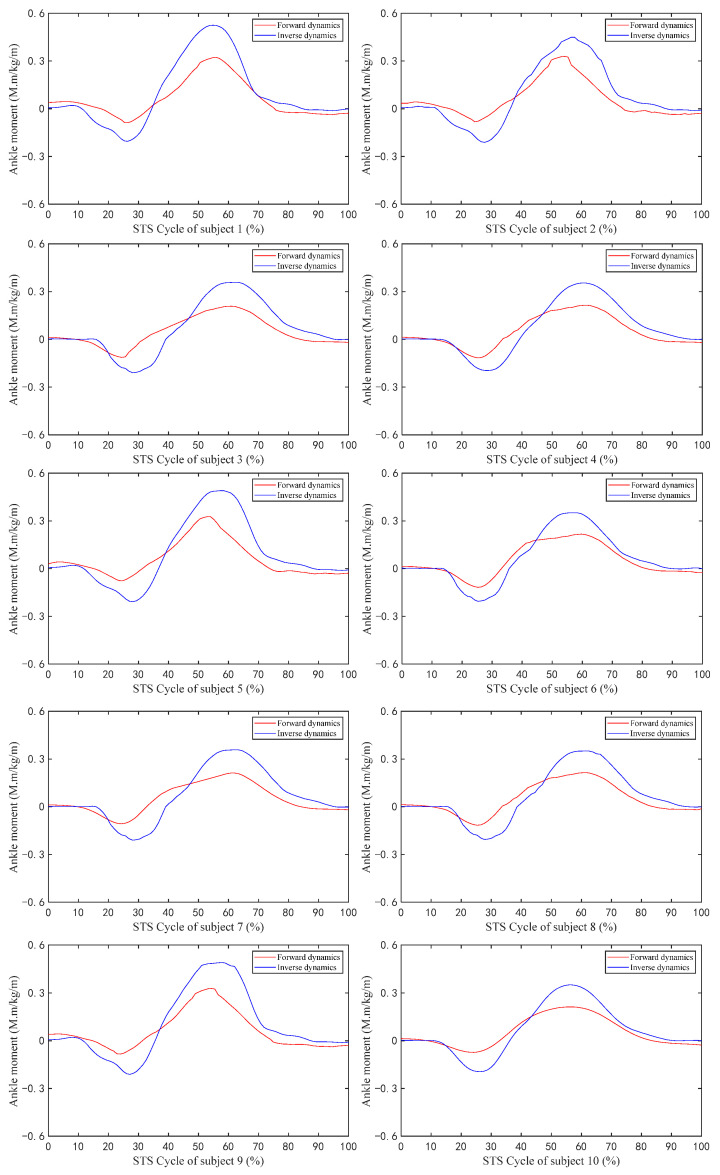
The ankle joint moment of ten subjects during the STS process calculated by forward dynamics and inverse dynamics.

**Figure 9:**
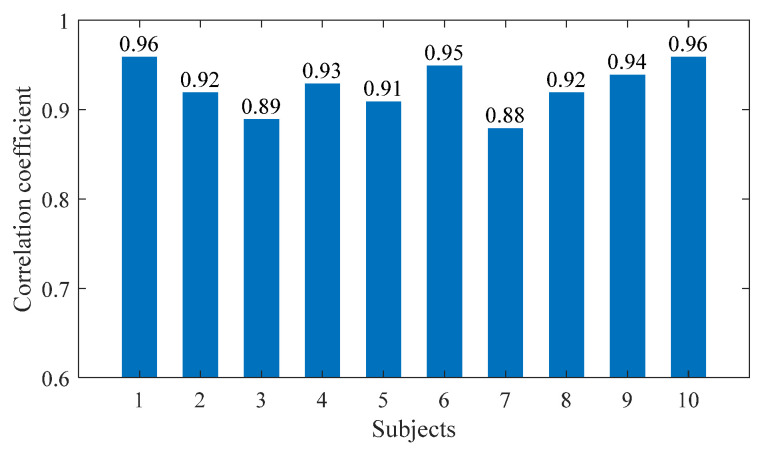
The correlation coefficients of the ankle joint moments of 10 subjects.

Figure 6 shows the changes in the values of the sEMG signal amplitude, activation degree, muscle fiber length, muscle fiber expansion and contraction speed, pennation angle, sagittal plane angle, muscle moment arm in the sagittal plane, and the muscle force in the sagittal plane, during the STS process of Subject 1. Figure 7 shows the changes in the average values of the joint angle, angular velocity, angular acceleration, and vertical chair reaction force, during the STS process of Subject 1. Figure 8 shows the ankle joint moments of ten subjects calculated using the forward dynamics and inverse dynamics methods, during the STS process. Figure 9 shows the correlation coefficients of the ankle joint moments of ten subjects calculated using the forward dynamics and inverse dynamics methods, and their average correlation coefficient is 0.926.

## 5. Discussion

In the STS process, the nervous system excites relevant muscles to develop active tension, which in turn drives the ankle joint to perform extension or flexion movements. As shown in Figure 6g,h, as the STS movement progresses, the corresponding muscle moment arm and muscle force of the four groups of muscles in the sagittal plane are time varying. For the tib-ant and ext-dig, their muscle moment arm and muscle force in the early phase of the STS movement are both greater than those in the latter. However, for the med-gas and soleus, the situation is approximately the opposite; the time when their muscle moment arm and muscle force start to increase is delayed compared with those of the tib-ant and ext-dig, that is to say, the muscle moment arm and muscle force of med-gas and soleus basically start to increase at the time when those of tib-ant and ext-dig start to decrease. Because, in the early stage of the STS process, the tib-ant and ext-dig are active muscles, while the med-gas and soleus are antagonistic muscles, and in the later stage of the STS process the situation is approximately opposite, the tib-ant and ext-dig convert to antagonistic muscles, while the med-gas and soleus convert to active muscles. In the whole STS process, the active muscle collaborates with the antagonistic muscle, where the active muscle provides power for ankle movement and the antagonistic muscle ensures stable ankle movement. Moreover, it can be observed that when the muscle is an active muscle, its muscle moment arm is greater than when it is an antagonistic muscle, which helps to generate greater torque on the ankle joint for the desired motion. Compared with Figure 6g,h in the whole STS process, the change trend of muscle activation is basically the same as that of muscle force in the sagittal plane, which are positively correlated. In addition, as shown in Figure 6c–f, the muscle fiber length, the muscle fiber velocity, the pennation angle, and the sagittal angle of the tib-ant and ext-dig have the same change trend because they are both cross ankle muscles and are both located in front of the ankle joint, resulting in consistent changes with the knee joint angle. Similarly, the med-gas and soleus, which are both located in the back of the ankle joint, should be the same, but as shown in the Figure 6, the muscle fiber length, the muscle fiber velocity, the pennation angle, and the sagittal angle of the med-gas and soleus have different change trends because the med-gas crosses both the ankle and knee joints but the soleus crosses only the ankle joint, resulting in inconsistent changes with different joint motions. Thus, it is clear that the changes in the parameters of muscles during the process of exerting force are most closely related to their attachment position on the bone and the joints they cross.

During the STS process, active muscle force serves as the driving force to overcome the body’s gravity and to do work, driving the body from a sitting position to a standing position. As shown in Figure 7a–c, the angle, the angular velocity, and the angular acceleration of the hip changes sharply prior to those of the knee and ankle, which indicates that the HAT of the human bend forward first to lead the center of mass of the human body upon the feet, at the same time, the buttocks begin to leave the seat, and the knee and ankle joints begin to move in sequence, and each limb of the body starts to stretch and complete the process from sitting to standing up until standing upright.

As shown in Figure 8, all the change trends of ankle joint moments calculated using both the forward dynamics and the inverse dynamics methods are the same, but the maximum and minimum values calculated using the inverse dynamics method are both greater than those calculated by the forward dynamics method, because the ankle joint musculoskeletal information for forward dynamics in this paper only contains four main muscles (tib-ant, ext-dig, med-gas, and soleus) which are the main muscles but not all of the ankle muscles participating in the flexion and extension movements of the ankle joint and contributing to the ankle joint moment. 

As shown in Figure 9, the correlation coefficients calculated for all subjects in this article are within the strong correlation range. The high correlation coefficient between the two methods’ results indicates that both methods are available for calculating ankle joint moment. However, in slow STS motion, the lower limb muscles move slowly, making it easier to collect sEMG signals. The forward dynamics method based on sEMG signals can calculate ankle joint moment, as well as muscle forces, helping doctors to better evaluate patients’ physical conditions. In STS motion, sEMG signals change rapidly, and the difficulty of signal measurement and processing increases relatively. At this time, it is more difficult to calculate joint moment using the forward dynamics method, while calculating joint moment using the inverse dynamics method is not greatly affected by STS speed. Therefore, suitable methods can be selected according to different needs.

In addition, as shown in Figure 9, the correlation coefficients of the joint moment results calculated using the two methods for ten subjects are different, because the ten subjects not only had different quantifiable physical characteristics (age, height, and weight), but also had completely different standing habits and speed each time. Especially when the standing speed is slow and the smoothness of the entire process is poor, particularly when there are jerky movements in the middle, the muscle force needs to be strong but accompanied by obvious shaking, which leads to significant changes in the sEMG signal and an increase in measurement error, resulting in a decrease in the accuracy of the calculation results. Therefore, the correlation coefficient results calculated through the inverse dynamics method are poor, as is the case with Subjects 3 and 7, and the correlation coefficients of Subjects 1 and 10 are relatively high, precisely because the STS process has more fluent movements and less muscle shaking. 

## 6. Conclusions

Calculating ankle joint moment and muscle force during the STS process is very important for evaluating human health and controlling rehabilitation equipment. In this paper, the musculoskeletal information of the ankle joint was established, and a self-developed wearable sensor system was used to collect the STS motion information of 10 subjects. The ankle joint moment was calculated using the forward dynamics and inverse dynamics methods. The results calculated by the two methods were very similar, with an average correlation coefficient of 0.926. The results show that it is feasible to calculate muscle force and ankle joint moment using the forward dynamics method. It provides a basis for doctors to evaluate patients’ STS rehabilitation conditions using non-invasive means.

In follow-up research work, we aim to continue to discuss the application of sEMG signals to calculate ankle joint moment in the STS process based on various factors such as gender, age, different pathological states, to increase the number of female or elderly subjects, and to analyze the different effects of individual differences. Furthermore, we plan to establish a model for musculoskeletal information that includes a bionic ankle and knee joint and more muscles, to investigate more accurate non-invasive methods for estimating joint moments and corresponding muscle forces. 

## Figures and Tables

**Table 1:** The ankle muscle parameters of Subject 1.

Muscle	Tib-Ant	Ext-Dig	Med-Gas	Soleus
Peaking force (N)	831	470	1432	3262
Optimal fiber length (mm)	93	96	57	47
Tendon length (mm)	211	325	368	236
Pennation angle at the optimum muscle fiber length (r)	0.087	0.140	0.300	0.436
Muscle origin (mm)	(17, 253, 10.4)	(2.8, 275.7, 26.4)	(−2.8, 17, −22.6)	(−1.8, 252, 6.7)
Via point 1 (mm)	(31, 33, −17)	(27.3, 27.3, 6.6)	(−13, 11.3, −24.5)	-
Via point 2 (mm)	-	(40.6, −2.8, 7.5)	-	-
Muscle insertion (mm)	(64, −22.7, −21.7)	(164, −41, 32.1)	(−46, −10.4, 2.8)	(−46, −10.4, 2.8)

Tib-ant only needs one via point; ext-dig needs two via points; med- gas only needs one via point; soleus does not need a via point; med-gas’s origin and via point 1 are located in the knee coordinate system; other points are located in the ankle coordinate system.

**Table 2:** The inertia parameters of human body segments of Subject 1.

Segments	Segment Length/ Height (%)	Segment Mass/ Whole Body Mass (%)	Center of Mass/ Segment Length Distance	Moment of Inertia (kg·m^2^)
Foot	14.77	3.6	0.5	0.0044
Shank	23.86	10.6	0.567	0.0385
Thigh	28.13	22.7	0.567	0.1987
HAT	50.17	63.1	0.374	0.9180

## Data Availability

Data are unavailable due to privacy or ethical restrictions.
